# VINA-SLAM: A Voxel-Based Inertial and Normal-Aligned LiDAR–IMU SLAM

**DOI:** 10.3390/s26061810

**Published:** 2026-03-13

**Authors:** Ruyang Zhang, Bingyu Sun

**Affiliations:** 1 School of Artificial Intelligence, Anhui University of Science and Technology, Huainan 232001, China; zry1286631670@gmail.com; 2 Hefei Institute of Intelligent Machines, Chinese Academy of Sciences, Hefei 230031, China

**Keywords:** LiDAR SLAM, voxel normal consistency, degenerate scenes, bundle adjustment

## Abstract

Environments with sparse or repetitive geometric structures, such as long corridors and narrow stairwells, remain challenging for LiDAR–inertial simultaneous localization and mapping (LiDAR–IMU SLAM) due to insufficient geometric observability and unreliable data associations. To address these issues, we propose VINA-SLAM, a novel LiDAR–IMU SLAM framework that constructs a unified global voxel map to explicitly exploit structural consistency. VINA-SLAM continuously tracks surface normals stored in the global voxel map using a normal-guided correspondence strategy, enabling stable scan-to-map alignment in degenerate scenes. Furthermore, a tangent-space metric is introduced to supplement missing rotational constraints around planar regions, providing reliable initial pose estimates for local optimization. A tightly coupled sliding-window bundle adjustment is then formulated by jointly incorporating IMU factors, voxel normal consistency factors, and planar regularization terms. In particular, the minimum eigenvalue of each voxel’s covariance is used as a statistically principled planar constraint, improving the Hessian conditioning and cross-view geometric consistency. The proposed system directly aligns raw LiDAR scans to the voxelized map without explicit feature extraction or loop closure. Experiments on 25 sequences from the HILTI and MARS-LVIG datasets show that VINA-SLAM reduces ATE by 25–40% on average while maintaining real-time performance at 10 Hz in the evaluated geometrically degenerate environments.

## 1. Introduction

Simultaneous localization and mapping (SLAM) [1] provides a fundamental service of robust autonomous navigation and perception for intelligent devices, which typically rely on accurate environmental modeling and localization. Benefiting from the continuous reduction in sensor cost and the ability of LiDAR to provide dense, active, and high-precision depth measurements, LiDAR-based localization and mapping techniques have been widely applied in the field of robotics [2,3]. Compared with traditional GNSS-based [4,5] and visual-based [6,7] systems, LiDAR localization often shows higher reliability in indoor, underground, and occluded environments. Moreover, in tasks that demand high localization accuracy, such as safe obstacle avoidance and precise path following, SLAM often acts as a practical core component of robotic navigation pipelines.

In real-world scenarios, intelligent robots often operate in semi-enclosed man-made environments such as building corridors, stairwells, long planar facades, and repetitive structural areas. These scenes are degenerate for traditional LiDAR SLAM systems [8] because they offer limited diversity in observation directions despite strong geometric regularities. However, due to the limited diversity of observation directions, the point-to-plane constraints, first introduced in the Chen–Medioni point-to-plane ICP framework [9], become overly sensitive to translational estimation while providing insufficient rotational observability around the plane normal. As a result, the system may suffer from unobservable orientations and incorrect data associations. In such environments, the lack of informative geometric structure and sparse feature correspondences leads to ill-conditioned Hessian (information) matrices in point-to-plane optimization, which has been extensively analyzed in recent degeneracy-aware LiDAR registration works [10,11,12,13]. Moreover, high-speed motion and the resulting motion distortion further weaken data associations and aggravate drift in LiDAR-inertial odometry [14]. Although existing LIO and LOAM frameworks achieve considerable robustness through explicit extraction of edge and planar features (e.g., [15,16,17,18]), they still face fundamental limitations in degenerate environments, where insufficient rotational observability, degraded Hessian conditioning, and weak cross-temporal geometric consistency remain challenging bottlenecks.

To address these challenges, this paper proposes a tightly coupled LiDAR–IMU framework centered on *voxel normal consistency* (VNC), which enhances system performance through a unified geometric representation. Specifically, without introducing additional sensors or computational overhead, the system employs an adaptive voxel map as a global geometric carrier. On the front end, both point-to-plane and normal-consistency observations are directly registered to the global voxel map, explicitly reinforcing rotational observability and improving the robustness of the front end in degenerate environments. On the back end, a sliding-window local bundle adjustment introduces a regularization term based on the minimum eigenvalue of the voxel covariance, $\lambda_{\min}$, to improve the Hessian condition number, while a tightly coupled integration of normal-alignment and IMU preintegration factors ensures long-term consistency and numerical stability. The main contributions of this paper are summarized as follows:

- **VNC-based Front-end Constraint:** We propose a novel observation model based on *voxel normal consistency* (VNC). By aligning the surface normals of the scan frames with the global voxel normals in the tangent space, the method explicitly strengthens rotational observability under highly dynamic and degenerate conditions, improving front-end rotation estimation in the evaluated scenarios (see Section 4).
- **Statistically Driven Local BA:** A *voxel minimum eigenvalue* ($\lambda_{\min}$) is introduced as a planarity regularization term in the sliding-window BA, which improves the Hessian condition number. Moreover, the normal-alignment factor provides inter-frame rotational priors (see Section 5). Its tight coupling with IMU preintegration improves the numerical stability and information fusion capability of the back end.
- **Unified Voxel Map and Tightly Coupled Implementation:** A unified adaptive voxel map is shared between the front and back ends, reducing representational discrepancy and information loss. Through tight coupling with IMU preintegration, the system achieves consistent mapping and real-time performance without relying on loop closure (see Section 3).
- **Comprehensive Validation and Reproducibility:** Extensive evaluations are conducted on 25 representative sequences from the HILTI 2022 and 2023 datasets and the MARS-LVIG dataset. Compared with existing open-source approaches, the proposed method yields lower ATE on most evaluated sequences while maintaining deployable latency and memory usage (see Section 6). The source code and configurations will be released in accordance with journal policies to support reproducibility.

**Figure 1:**
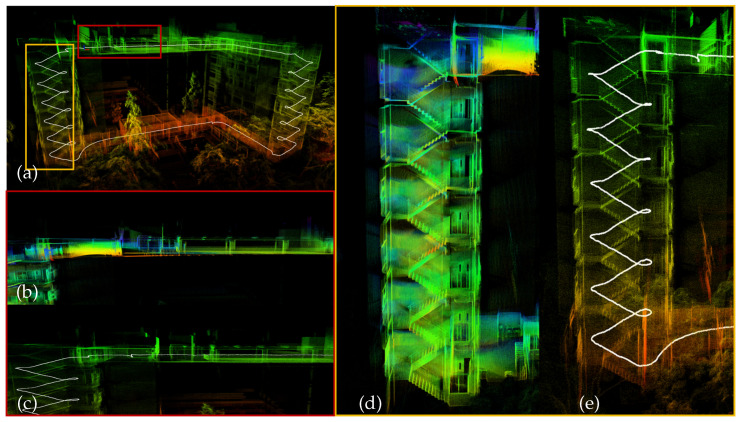
Example mapping results and local comparisons. (**a**) Global map reconstructed by **VINA-SLAM** with two marked regions: the red box and the yellow box. (**b**) Zoom-in of the red region reconstructed by **FAST-LIO2**. (**c**) Zoom-in of the same red region reconstructed by **VINA-SLAM**. (**d**) Zoom-in of the yellow region reconstructed by **FAST-LIO2**. (**e**) Zoom-in of the same yellow region reconstructed by **VINA-SLAM**, showing clearer planar structures and improved consistency.

Experimental results show that the proposed method reduces trajectory errors and yields more consistent convergence in typical degenerate scenarios such as narrow corridors, stairwells, repetitive structures, and high-speed maneuvers. As illustrated in Figure 1, our method reconstructs clearer planar structures and yields more consistent local geometry compared with FAST-LIO2. With a 10 Hz LiDAR input, the system maintains real-time performance and low memory consumption, while achieving consistent mapping without relying on loop closure. These results indicate that the joint modeling of voxel normal consistency and statistically regularized BA improves rotational observability, numerical conditioning, and cross-temporal geometric consistency, and provides a basis for incorporating global loop closure and large-scale consistent mapping in future work.

## 2. Related Works

### 2.1. LiDAR(-Inertial) Odometry and SLAM

At present, most 3D LiDAR-SLAM systems are built upon the LOAM framework [15], which mainly consists of three modules: feature extraction, odometry, and mapping. In the feature extraction stage, edge and planar points are identified based on the local smoothness of the point cloud, and these feature points are subsequently used for pose estimation to improve computational efficiency. To mitigate long-term drift, LeGO-LOAM [19] introduces ground segmentation and loop closure mechanisms, further enhancing overall system performance. Subsequent extensions, such as LOAM-Livox [20], R-LOAM [21], and F-LOAM [22], focus primarily on improving computational efficiency and robustness.

Integrating an IMU into the SLAM framework significantly enhances the accuracy and robustness of odometry, playing a crucial role in compensating for motion distortion during LiDAR scanning. The high-frequency IMU data provides more accurate initial pose estimates for ICP, thereby improving both convergence and final registration quality. LION [23] adopts a loosely coupled optimization approach to process point cloud data, while LIO-SAM [24] tightly couples IMU and LiDAR measurements within a factor graph, achieving improved accuracy and convergence at the cost of higher computational complexity when handling large-scale data. To further improve system efficiency, LiLi-OM [25] introduces a smaller optimization window.

LINS [26] introduces a tightly coupled iterative Kalman filter into the LIO framework, leveraging the Kalman gain formulation to reduce computational complexity from the measurement dimension to the state dimension. FAST-LIO [16] proposes a novel Kalman gain formulation and optimizes point cloud registration using an incremental k-d tree, further alleviating computational burden. FAST-LIO2 [17] improves the incremental k-d tree structure, eliminating the need for explicit feature extraction and enhancing overall efficiency. Faster-LIO [18] introduces parallel sparse incremental voxels to further accelerate processing speed. Point-LIO [27], built upon FAST-LIO2, incorporates IMU saturation checks to improve robustness against mechanical vibrations.

Most existing LIO methods rely primarily on point-to-plane residuals for pose estimation [16,17,18,23,25,26]; however, in degenerate environments such as long corridors, narrow stairwells, and repetitive structural regions—where strong geometric priors exist but observation directions are limited—they still suffer from significant deficiencies [11]. In particular, under high-speed motion and sparse measurement conditions, insufficient rotational observability, degraded numerical conditioning, and weak long-term consistency become especially pronounced [14].

In contrast, visual SLAM methods have demonstrated unique advantages in addressing similar challenges, particularly in indoor environments. Since drift in SLAM systems is largely caused by inaccurate rotation estimation [28,29,30], visual SLAM enhances robustness in low-texture scenes by leveraging the geometric structure of the environment. For instance, Structure-SLAM [31] utilizes line features and surface normals combined with spherical mean-shift clustering under a weak Manhattan-world assumption to estimate drift-free rotation, thereby improving pose estimation robustness. Similarly, ManhattanSLAM [32] constructs a Manhattan map to detect and track Manhattan frames, decoupling pose estimation and providing drift-free rotational constraints, thus enabling robust tracking in challenging scenes and further enhancing the stability of visual SLAM in complex indoor environments.

Although these visual SLAM methods have demonstrated outstanding performance in indoor environments, the point-to-plane residual remains the dominant approach for pose estimation in LiDAR-based SLAM. Inspired by the advantages of visual SLAM in handling complex environments, VINA-SLAM introduces an explicit rotational normal constraint to effectively enhance rotational observability. This innovation mitigates the adverse effects of high-speed motion and sparse measurements, further improving the robustness and accuracy of LiDAR-based SLAM systems.

### 2.2. LiDAR Point Cloud Registration Strategies

Point cloud registration is one of the key techniques in SLAM. Traditional registration methods such as ICP and GICP [33,34] have been widely adopted; however, these approaches are prone to cumulative errors, leading to noticeable drift during long-term operation. To alleviate this issue, researchers have introduced the bundle adjustment (BA) framework for global optimization. BALM [35] unifies point-to-plane and point-to-line registration constraints into a BA formulation, significantly reducing drift and improving global consistency. BALM2 [36] further accelerates computation by introducing point clustering, thereby enhancing overall efficiency.

π-LSAM [37] incorporates planar geometric priors commonly observed in indoor environments by modeling planes as explicit landmarks, thereby enhancing robustness in low-texture conditions. EigenFactor [38] proposes a two-layer optimization framework that pre-aggregates point cloud statistics for each plane, improving computational efficiency. As scene complexity and data scale increase, the computational burden of factor-graph optimization grows rapidly. BALM3 [39] introduces a Majorization–Minimization (MM) framework that decomposes the original problem into a series of convex subproblems, effectively accelerating computation while ensuring global consistency.

VINA-SLAM integrates both pairwise and multi-view registration strategies. In the odometry stage, an efficient planar map (VoxelMap) is employed for scan-to-map registration. For local mapping, the framework follows the philosophy of the BALM series by performing LiDAR–Inertial bundle adjustment (BA) optimization, achieving a balance between efficiency and accuracy while enhancing robustness, particularly in low-texture environments.

## 3. System Overview

**Figure 2:**
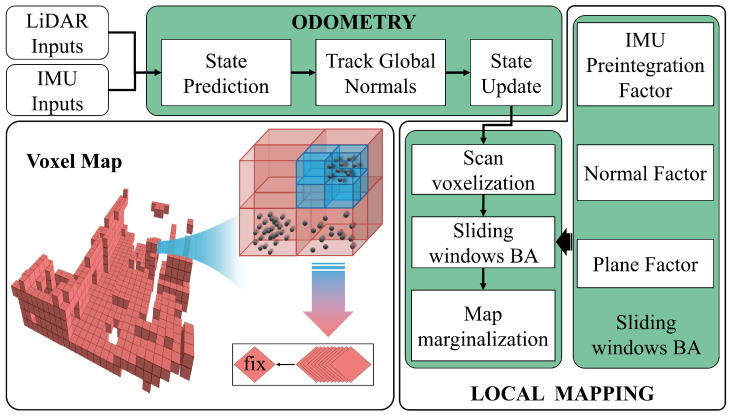
Architecture of the method. The IMU and LiDAR inputs are fed into the system. In the forward propagation phase, IMU measurements are integrated to recursively update the current state. The front-end module (see Section 4) is responsible for continuously tracking global normal vectors and incorporating them into the pose optimization framework. In the local mapping phase (see Section 5), the tracked global normal vectors are integrated into the Local BA to ensure accurate map registration. Finally, the oldest scan data within the sliding window will be marginalized and registered into the global map.

The overall workflow of VINA-SLAM is illustrated in Figure 2. First, based on IMU preintegration, the system provides motion priors for LiDAR points sequentially sampled within each scan period, compensating for motion distortion and reconstructing a temporally consistent point cloud. We define the system state $x_{i}$ of the IMU in the world frame at the end of the *i*-th LiDAR scan as

(1) $$x_{i}=\left[R_{i},p_{i},v_{i},b_{i}^{g},b_{i}^{a}\right]$$

where $R_{i}\in SO\left(3\right)$, $p_{i}\in R^{3}$, and $v_{i}$ denote the orientation, position, and velocity of the IMU in the world frame, respectively, while $b_{i}^{g}$ and $b_{i}^{a}$ represent the gyroscope and accelerometer biases in the local frame.

The corrected point cloud is then projected into the world frame according to the IMU-predicted pose and voxelized using the voxel size of the current global map. Each voxel is analyzed via principal component analysis (PCA) and spectral decomposition to determine whether it represents a planar surface, from which the corresponding normal vector is extracted. Both the global map voxels and the projected scan voxels are managed using a hash table, ensuring that voxels sharing the same spatial coordinates in the world frame possess identical hash keys, thereby enabling efficient normal-vector lookup. To ensure reliable matching, voxels containing fewer than $N_{min}=20$ points are excluded from planar fitting.

The odometry module performs real-time state estimation—including pose and velocity—by tightly fusing point-to-plane constraints, normal-vector consistency constraints, and IMU measurements within an extended Kalman filter (EKF) framework (red dashed box, see Section 4). After the current pose is estimated, the corresponding scan is inserted into a sliding window. Within this window, the system continues to track global normals following the same procedure and performs LiDAR–inertial joint bundle adjustment (BA) to simultaneously optimize all states and the local map (blue dashed box, see Section 5). The oldest keyframe is subsequently marginalized, and the global voxel map is updated, achieving continuous and consistent mapping and localization.

## 4. Front-End LIO with Normal Consistency Constraint

Conventional LIO systems typically perform registration updates by minimizing the Euclidean point-to-plane distance [15,17]. However, in plane-dominant environments such as building facades, corridors, and ground surfaces, the single point-to-plane constraint provides insufficient rotational observability around the plane normal. This often leads to attitude drift under pure translation, rotation about the normal direction, or planar degeneracy. Inspired by the concept of structured constraints [31,32,40], we explicitly introduce a *normal consistency* factor in the front end. This factor acts solely on the rotational degrees of freedom and complements the point-to-plane constraint, thereby enhancing attitude observability and overall robustness in degenerate geometries.

### 4.1. Surface Normal Tracking

**Figure 3:**
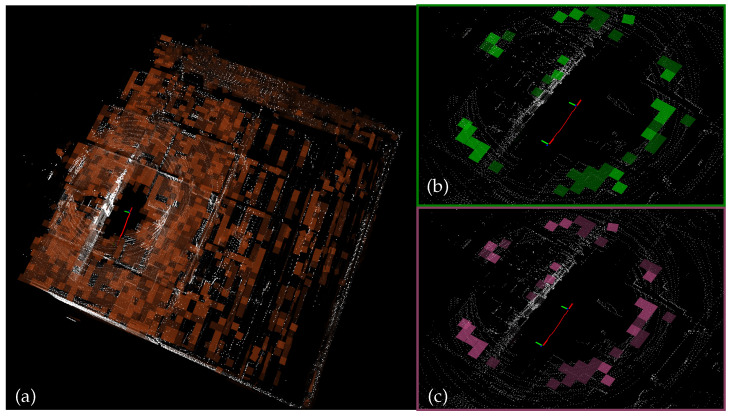
Voxel normal tracking. (**a**) Orange: planar voxels in the global map; (**b**) Green: planar voxels detected in the current scan frame; (**c**) Pink: one-to-one paired map voxels corresponding to scan voxels.

To obtain stable rotational observations, VINA-SLAM performs global normal tracking on voxel grids that share identical resolutions and hash keys between the scan frame and the global map (Figure 3). The process consists of four stages: temporal alignment and world projection, voxelization and planar detection, normal and uncertainty estimation, and hash-based pairing with angular gating.

#### 4.1.1. Temporal Alignment and World Projection

For LiDAR points sequentially sampled within a single scan period, the prior pose (R,p) is obtained through IMU preintegration to correct motion distortion and reconstruct a temporally consistent scan. The corrected point $p_{i}$ is then projected into the world frame as:

(2) $$p_{i}^{w}=R\;p_{i}+p.$$

#### 4.1.2. Spatial Voxelization and Planar Detection

The corrected points $\left\{p_{i}^{w}\right\}$ are voxelized with a voxel size of *s*, and voxel blocks are accessed via a hash table in O(1) complexity. For each voxel V, we accumulate the number of samples *N*, centroid $\bar{p}$, and covariance matrix:

$$\bar{p}=\frac{1}{N}\;\sum_{p\in V}p,\;C=\frac{1}{N}\;\sum_{p\in V}\left(p-\bar{p}\right){\left(p-\bar{p}\right)}^{⊤},$$

and perform eigen-decomposition $C=U\;\mathrm{diag}\left(\lambda_{1},\lambda_{2},\lambda_{3}\right)U^{⊤}$ ($\lambda_{1}\;\leq \;\lambda_{2}\;\leq \;\lambda_{3}$). To ensure high-quality matching, voxels with $N<N_{\min}\left(=20\right)$ are discarded. A dual-threshold criterion is used to identify planar voxels:

(3) $$\lambda_{1}<\lambda_{\min}^{\mathrm{abs}}\;\mathrm{and}\;\lambda_{1}/\lambda_{3}<\tau_{\mathrm{plane}},$$

where $\lambda_{\min}^{\mathrm{abs}}=0.0025$ and $\tau_{\mathrm{plane}}=0.25$, ensuring both a noise lower bound and relative flatness.

#### 4.1.3. Normal and Uncertainty Estimation

For planar voxels, the normal vector is defined as the eigenvector corresponding to the smallest eigenvalue, $n=u_{1}$ (∥n∥=1), and the plane center is set to the centroid $\bar{p}$. Based on eigenvalue perturbation analysis, the sensitivity of the smallest eigenvector with respect to the covariance matrix can be approximated as

(4) $$\frac{\partial u_{1}}{\partial C}\;\approx \;\sum_{k=2}^{3}\frac{u_{k}\;u_{k}^{⊤}\;u_{1}\;u_{1}^{⊤}+u_{1}\;u_{k}^{⊤}\;u_{k}\;u_{1}^{⊤}}{\lambda_{1}-\lambda_{k}},$$

from which a first-order approximation of the normal covariance is obtained as $\Sigma_{n}\approx J\;\mathrm{Cov}\left(C\right)\;J^{⊤}$, used for subsequent uncertainty weighting.

#### 4.1.4. Hash-Based Pairing and Angular Gating

The scan voxel map and the global voxel map share the same resolution and hash key structure, such that voxels with identical indices (i,j,k) in the world frame directly form candidate pairs. For each candidate voxel pair with normals $\left(n_{\mathrm{scan}},n_{\mathrm{map}}\right)$, a sign disambiguation is first performed (if the inner product <0, the normal is negated), followed by angular gating:

(5) $$\arccos\;\left(n_{\mathrm{scan}}^{⊤}n_{\mathrm{map}}\right)\leq \theta_{\max}\;\left(10^{\circ }\right).$$

The surviving “center–normal” observation pairs $\left({\bar{p}}_{\mathrm{scan}},n_{\mathrm{scan}}\right)$ and $\left({\bar{p}}_{\mathrm{map}},n_{\mathrm{map}}\right)$, along with their corresponding uncertainties, are preserved for use in the front-end EKF and back-end BA optimization.

### 4.2. Normal Consistency Residual and EKF Update

#### 4.2.1. State and Propagation

At the end of the *i*-th frame, the system state is defined as $x_{i}=\left[R_{i},p_{i},v_{i},b_{i}^{g},b_{i}^{a}\right]$, where $R_{i}\;\in \;\mathrm{SO}\left(3\right)$, $p_{i},v_{i}\;\in \;R^{3}$, and $b_{i}^{g},b_{i}^{a}$ denote the gyroscope and accelerometer biases, respectively. The IMU preintegration [41] is employed to propagate the state from frame *i* to i+1, yielding the predicted mean ${\hat{x}}_{i+1}^{-}$ and covariance $P_{i+1}^{-}$.

#### 4.2.2. Normal Consistency Residual in the Tangent Space

Given the prior attitude ${\hat{R}}_{i+1}^{-}$, the normal of the scan is rotated into the world frame, and the consistency is measured in the tangent space of $n_{\mathrm{map}}$:

(6) $$\begin{array}{cc} {\tilde{n}}_{\mathrm{scan}} & ={\hat{R}}_{i+1}^{-}n_{\mathrm{scan}},\\ r_{n} & =\Pi_{n_{\mathrm{map}}}\left({\tilde{n}}_{\mathrm{scan}}-n_{\mathrm{map}}\right),\\ \Pi_{n} & =I-nn^{⊤}. \end{array}$$

The Jacobian with respect to the small rotational perturbation δθ is

(7) $$J_{n,R}=\Pi_{n_{\mathrm{map}}}{\left[{\hat{R}}_{i+1}^{-}n_{\mathrm{scan}}\right]}_{\times },$$

while the Jacobians with respect to $p,v,b^{g},b^{a}$ are all zero. The residual covariance is defined as

(8) $$\Sigma_{r}=\Pi_{n_{\mathrm{map}}}\left(\Sigma_{n,\mathrm{scan}}+\Sigma_{n,\mathrm{map}}\right)\Pi_{n_{\mathrm{map}}}^{⊤}.$$

#### 4.2.3. Joint Update with Point-to-Plane Constraint

The point-to-plane residual is given by

(9) $$r_{p}=n_{\mathrm{map}}^{⊤}\left({\hat{R}}_{i+1}^{-}p_{s}+{\hat{p}}_{i+1}^{-}-{\bar{p}}_{\mathrm{map}}\right),$$

with its Jacobians expressed as $\partial r_{p}/\partial \delta \theta =n_{\mathrm{map}}^{⊤}{\left[{\hat{R}}_{i+1}^{-}p_{s}\right]}_{\times },\;\partial r_{p}/\partial \delta p=n_{\mathrm{map}}^{⊤},$ and zeros for the remaining terms. Stacking the normal and point-to-plane measurements yields

(10) $$\begin{array}{cc} r & =\left[\left\{r_{n}\right\};\left\{r_{p}\right\}\right],\\ H & =\left[\left\{J_{n,R}\;\;0\right\};\left\{J_{p}\right\}\right],\\ R & =\mathrm{blkdiag}(\left\{\Sigma_{r}\right\},\left\{\sigma_{r_{p}}^{2}\right\}). \end{array}$$

The EKF update is then performed as

(11) $$\begin{array}{cc} S & =HP_{i+1}^{-}H^{⊤}+R,\\ K & =P_{i+1}^{-}H^{⊤}S^{-1},\\ \delta x & =Kr,\\ P_{i+1}^{+} & =\left(I-KH\right)P_{i+1}^{-}. \end{array}$$

The attitude is updated via right-multiplicative exponential mapping $R_{i+1}^{+}=R_{i+1}^{-}\exp\left(\delta \theta \right)$, while the remaining state components are updated linearly to obtain the posterior estimate ${\hat{x}}_{i+1}^{+}$.

## 5. LiDAR-Inertial BA Optimization

In LiDAR scanning environments, geometric information along certain directions may be missing or weakened, leading to poor attitude observability and an ill-conditioned Hessian matrix, which in turn amplifies instability in pose estimation. To alleviate such degeneracies, joint optimization using multi-view observations has proven to be an effective strategy, typically formulated within the framework of bundle adjustment (BA). Conventional approaches often adopt a “two-layer” structure: relative poses between selected scan pairs are first estimated through pairwise registration, followed by global optimization at the pose-graph level. This decoupled strategy offers good computational efficiency for real-time SLAM systems; however, its representation of the original point cloud constraints is indirect, providing insufficient geometric “curvature” in degenerate dimensions. Consequently, when relying solely on relative pose constraints, the improvement in attitude observability remains limited.

Alternatively, directly incorporating all raw points and geometric features into the BA formulation can enhance constraint strength, but the computational complexity scales rapidly with the number of observations, making real-time operation infeasible. To balance geometric representation and computational efficiency, we adopt the concept of point clustering from the BALM series [35,36,39], and integrate IMU factors [41], planar factors [38], and normal-vector factors to construct a LiDAR–inertial bundle adjustment (BA) optimization. This formulation jointly estimates multiple states in parallel and, when necessary, includes gravity estimation within the optimization process.

### 5.1. State Definition

Assume that the sliding window contains *N* LiDAR frames, and the state corresponding to the *i*-th frame is defined in Equation (1).

The set of all optimization variables is expressed as

(12) $$\chi =\left[x_{1},\ldots ,x_{N},g\right],\;x_{i}=\left(R_{i},p_{i},v_{i},b_{i}^{g},b_{i}^{a}\right)$$

The gravity vector *g* is shared within the sliding window. To improve computational efficiency, *g* is only jointly optimized when the system observes *persistent* geometric degeneration during front-end pose estimation (see Appendix C).

### 5.2. BA Residual Function Modeling

**Figure 4:**
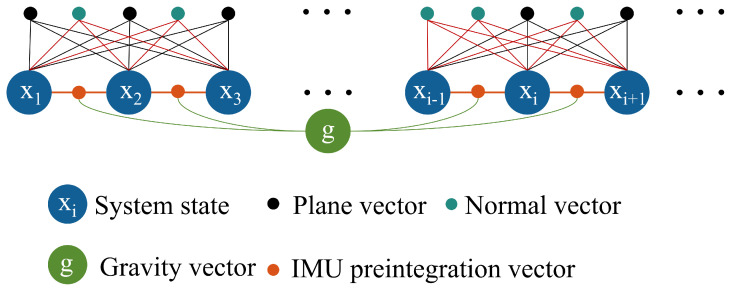
The factor graph representation of the proposed LiDAR-inertial bundle adjustment.

As illustrated in Figure 4, to enhance attitude observability and improve the Hessian condition number, the proposed optimization jointly incorporates IMU preintegration factors, planar factors, and normal-vector factors into an optimization function as follows:

(13) $$\begin{array}{c} \arg\min_{x}\;\left(\frac{1}{2}\sum_{i=1}^{N-1}{∥r_{i,i+1}\left(\chi \right)∥}_{\Sigma_{i,i+1}^{-1}}^{2}+\sum_{j=1}^{M}c_{j}\lambda_{j}^{\min}\left(\chi \right)\right.\\ \left.\;\;+\frac{1}{2}\sum_{j=1}^{M}\alpha_{j}{∥r_{j}^{n}\left(\chi \right)∥}^{2}\right) \end{array}$$

Here, $r_{i,i+1}\left(\chi \right)$ denotes the IMU factor, $\lambda_{j}^{\min}\left(\chi \right)$ the planar factor, and $r_{j}^{n}\left(\chi \right)$ the normal-vector factor.

#### 5.2.1. IMU Factor

The IMU factor $r_{i,i+1}\left(\chi \right)$ includes residuals of orientation, velocity, position, and bias, with the covariance $\Sigma_{i,i+1}$ derived from discrete noise propagation. The partial derivatives with respect to gravity are given by

(14) $$\begin{array}{cc} \frac{\partial r_{\Delta R_{ij}}}{\partial \delta g} & =0;\\ \frac{\partial r_{\Delta v_{ij}}}{\partial \delta g} & =-R_{i}^{⊤}\Delta t_{ij};\\ \frac{\partial r_{\Delta p_{ij}}}{\partial \delta g} & =-\frac{1}{2}R_{i}^{⊤}\Delta t_{ij}^{2}. \end{array}$$

#### 5.2.2. Planar Factor

Each LiDAR frame in the sliding window is transformed into the world frame using the current state $\left\{X_{i}\right\}$ as $p^{w}=R_{i}p^{b}+p_{i}$. Points are then grouped into voxels according to their world coordinates. For the *j*-th voxel, the aggregated statistics are computed with $N_{j}$ points, where $P_{j}=\sum p^{w}{p^{w}}^{⊤}$. The mean and covariance are thus given by:

(15) $${\bar{p}}_{j}=\frac{v_{j}}{N_{j}},\;C_{j}=\frac{P_{j}}{N_{j}}-{\bar{p}}_{j}{\bar{p}}_{j}^{⊤}.$$

Performing PCA on $C_{j}$ yields the ordered eigenvalues $\lambda_{0}^{\left(j\right)}\leq \lambda_{1}^{\left(j\right)}\leq \lambda_{2}^{\left(j\right)}$, where $\lambda_{\min}^{\left(j\right)}=\lambda_{0}^{\left(j\right)}$ characterizes the voxel thickness along the estimated normal direction; voxel *j* is incorporated into the back-end optimization if $N_{j}\geq N_{\min}^{\mathrm{BA}}$ (set to 5) and $\frac{\lambda_{0}^{\left(j\right)}}{\lambda_{1}^{\left(j\right)}}\leq \tau_{\mathrm{pl}}$ (with $\tau_{\mathrm{pl}}=0.12$). We use $N_{\min}=20$ in the front-end (Section 3 and Section 4) but $N_{\min}=5$ in the back-end, since multi-view aggregation over a sliding window of $N_{w}$ frames (where $N_{w}$ denotes the window size and is set to $N_{w}=10$ in our implementation) allows a voxel to accumulate on the order of $N_{\min}\;N_{w}$ points (i.e., ∼50 under our settings), which is typically sufficient for normal uncertainty convergence [42].

#### 5.2.3. Normal-Vector Factor

For the *j*-th voxel, the corresponding reference normal $n_{j}^{map}$ is retrieved from the global map. The smallest eigenvector $u_{0}^{\left(j\right)}$ is used to approximate the normal vector of the scan voxel, and the projection residual is defined as

(16) $$r_{j}^{n}=\left(I-n_{j}^{\mathrm{map}}{n_{j}^{\mathrm{map}}}^{⊤}\right)u_{0}^{\left(j\right)}.$$

The cost function in Equation (13) is optimized iteratively using the second-order Levenberg–Marquardt (LM) solver. To accelerate the optimization process, the analytical Jacobian and Hessian matrices of the cost function are derived in closed form. Detailed derivations of the Jacobian, Hessian, and solver implementation are provided in Appendix A.

## 6. Experimental Results

### 6.1. Datasets and Metrics

We evaluate the proposed system on two publicly available datasets with distinct characteristics: HILTI [43,44] and MARS-LVIG [45], comprising a total of 25 sequences (the complete sequence list is provided in Table A1). The HILTI dataset was collected in structured indoor and outdoor construction environments, using a Hesai XT-32 LiDAR (Hesai Technology, Shanghai, China) and a Bosch BMI085 IMU at 400 Hz (Robert Bosch GmbH, Stuttgart, Germany), and provides an official online evaluation server. The MARS-LVIG dataset, in contrast, was recorded by an unmanned aerial vehicle (UAV) equipped with a Livox Avia LiDAR (Livox Technology Company Limited, Shenzhen, China) with an integrated BMI088 IMU (Robert Bosch GmbH, Stuttgart, Germany) operating at 200 Hz, flying at an altitude of approximately 100 m. Across all sequences, the LiDAR sampling frequency is 10 Hz.

**Figure 5:**
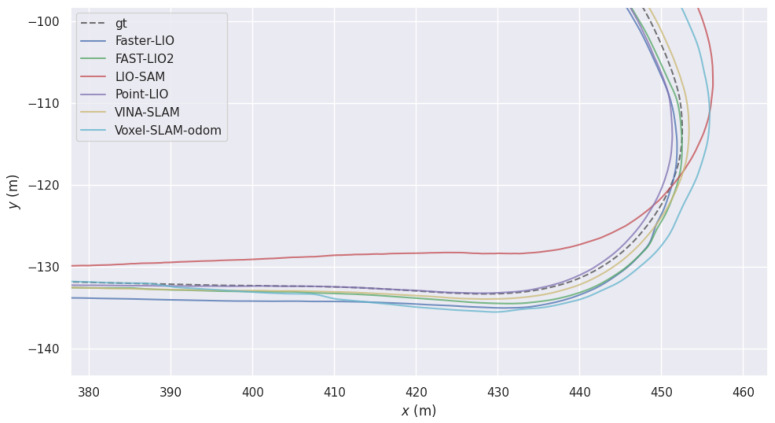
Local trajectory comparison in the degraded region of the MARS-LVIG Mars4-1 sequence.

All estimated trajectories are saved in TUM format. Temporal alignment is achieved through linear interpolation to match ground-truth timestamps, with a maximum time difference threshold of 50 ms. For HILTI, we follow the official online evaluation protocol for alignment and metric computation, and report the translation RMSE. For MARS-LVIG, we compute ATE (RMSE) using EVO [46] with SE(3) alignment enabled. The same evaluation protocol is used for the sequence-level cumulative drift curves reported later in Figure 5. In addition to ATE, we report the distance-normalized ATE (RMSE) $\epsilon_{\mathrm{km}}$ as a long-term drift indicator:

(17) $$\epsilon_{\mathrm{km}}\;\left(m/\mathrm{km}\right)\;=\;\frac{{\mathrm{ATE}}_{\mathrm{RMSE}}\;\left(m\right)}{L_{\mathrm{GT}}\;\left(\mathrm{km}\right)},$$

where $L_{\mathrm{GT}}$ denotes the traveled distance computed by cumulatively summing the ground-truth trajectory increments.

### 6.2. Baselines and Implementation Details

In this section, the proposed VINA-SLAM is compared against several representative open-source LiDAR (and LiDAR–Inertial) SLAM methods, including LeGo-LOAM [19], LiLi-OM [25], LINS [26], LIO-SAM [24], FAST-LIO2 [17], Faster-LIO [18], Point-LIO [27], and Voxel-SLAM [47]. For LeGo-LOAM [19], LiLi-OM [25], LINS [26], LIO-SAM [24], FAST-LIO2 [17], Faster-LIO [18], and Point-LIO [27], the results are taken directly from the public benchmark values reported in Voxel-SLAM [47]. The “odom” results of Voxel-SLAM [47] are reproduced and evaluated under the same experimental protocol for consistency. Unless otherwise specified, all baseline methods use their default parameters and official sensor configurations. We further conduct ablation studies to analyze the contribution of each module: Ours (Odom) represents the front-end odometry (point-to-plane constraint + normal consistency constraint), while Ours (Odom + BA) includes the local BA module on top of the front-end.

All experiments are conducted on the same desktop workstation equipped with an Intel i9-13900K CPU (32 threads, 5.5 GHz), 128 GB RAM, and dual NVIDIA GPUs (RTX 4070), running Ubuntu 24.04 LTS (Linux kernel 6.8.0). For the HILTI dataset, the voxel sizes for indoor and outdoor scenes are set to $L_{r}=1$ m and $L_{r}=4$ m, respectively, with a voxel downsampling resolution of $L_{d}=0.1$ m. For the MARS-LVIG dataset, $L_{r}=4$ m and $L_{d}=0.5$ m are used. The maximum map hierarchy level is $L_{\max}=3$. The minimum number of points for planar fitting is fixed at $N_{min}=20$, and the planarity criterion is $\frac{\lambda_{3}}{\lambda_{2}}<0.0625$, where $\lambda_{i}\;\left(i=1,2,3\right)$ are the eigenvalues of the covariance matrix satisfying $\lambda_{1}\leq \lambda_{2}\leq \lambda_{3}$. The window size for the local BA is set to 10.

### 6.3. Comparison on the HILTI Dataset

**Figure 6:**
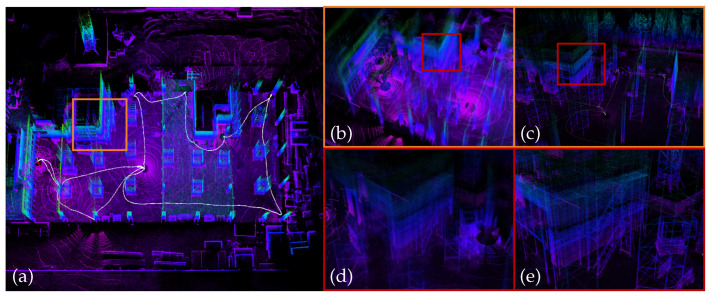
Overview of sequence “hilti01”: (**a**) Point cloud map constructed by VINA-SLAM. (**b**) Locally repetitive building components identified by VINA-SLAM. (**c**) Scaffolding regions prone to false associations detected by VINA-SLAM. (**d**) Locally repetitive building components identified by FAST-LIO2. (**e**) Scaffolding regions prone to false associations detected by FAST-LIO2.

**Figure 7:**
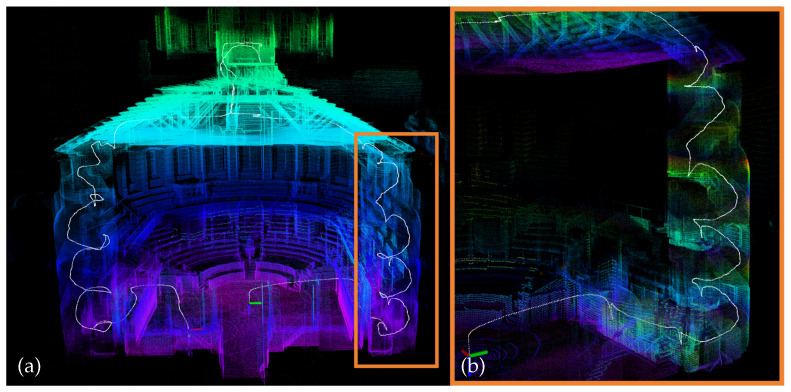
Overview of sequence “hilti05”: (**a**) Point cloud map constructed by VINA-SLAM. (**b**) The orange border indicates the trajectory within the narrow stairwell and its corresponding point cloud map.

The HILTI dataset targets realistic construction and building environments under GNSS-denied conditions, encompassing typical degenerate scenarios such as corridors, stairwells, open floors, pipe shafts, scaffolding, and metallic or glass reflective surfaces. The presence of numerous repetitive structures and reflective materials increases the likelihood of false data associations and outlier ratios, while narrow passages often induce tangential drift. Table 1 summarizes the translation RMSE results of all evaluated methods. Compared with systems that rely solely on explicit geometric feature extraction and matching, VINA-SLAM mitigates false associations in repetitive structures through its adaptive-resolution voxel map and normal-consistency constraints (see Figure 6), and reduces tangential drift in narrow corridors (see Figure 7). Figure 6 and Figure 7 are qualitative case visualizations with different focuses: Figure 6 highlights association ambiguity in repetitive or scaffolding regions and its mitigation by the voxel-map-based design, while Figure 7 illustrates trajectory and map consistency in a narrow stairwell scenario. Quantitative evidence is reported in Table 1 and Table 2, and the sequence-level drift curves in Figure 5. In most sequences, the inclusion of the local BA module in VINA-SLAM further reduces cumulative drift and achieves the best or second-best accuracy.

As shown in Table 2, VINA-SLAM reports lower distance-normalized errors on the HILTI dataset, with values ranging from 0.04 to 1.53 m/km. On the degenerate sequence hilti07, *Ours (odom + BA)* achieves 1.53 m/km, compared with 5.54 m/km (FAST-LIO2) and 3.46 m/km (Point-LIO).

### 6.4. Comparison on the MARS-LVIG Dataset

**Figure 8:**
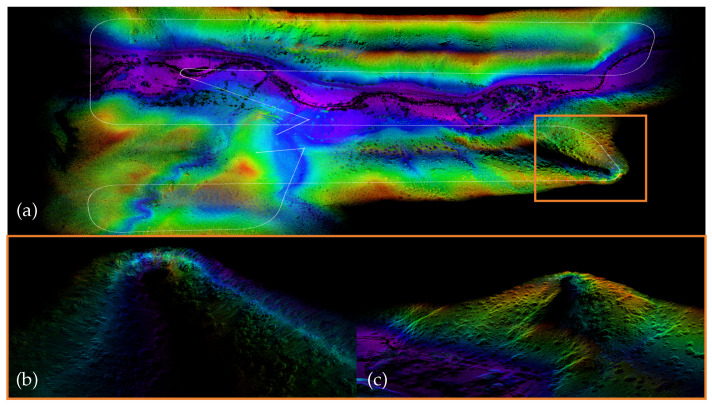
Overview of sequence “mars4-1”: (**a**) Point cloud map constructed by VINA-SLAM. (**b**) Orange box: degenerate region caused by sparse and long-range LiDAR returns, detected by FAST-LIO2. (**c**) Orange box: degenerate region caused by sparse and long-range LiDAR returns, detected by VINA-SLAM.

Unlike the HILTI dataset, which features small-scale, highly structured, and slow-moving indoor and construction environments, the MARS-LVIG dataset was collected in large-scale outdoor environments with flight speeds reaching up to 12 m/s, containing unstructured elements such as islands, hills, and rivers. Under such highly dynamic and long-range conditions, the local observability of point clouds becomes insufficient, and both IMU noise and temporal synchronization errors are magnified, degrading front-end registration accuracy. Nevertheless, the proposed method maintains consistent mapping behavior in degenerate regions (see Figure 8). This can be attributed to two main factors: first, the $\lambda_{\min}$ planarity term reduces “thickness” dispersion under sparse and long-range observations, improving the Hessian condition number; second, the normal-consistency constraint directly enhances rotational observability within the attitude subspace, mitigating heading drift during high-speed maneuvers. Furthermore, in locally degenerate scenes, the local BA provides inter-frame geometric aggregation, while IMU integration maintains local motion estimation, ensuring consistent map constraints over long trajectories. As summarized in Table 3, the proposed method shows competitive and often lower errors in unstructured and high-dynamic aerial scenarios.

As shown in Table 4, VINA-SLAM reports distance-normalized errors of 0.06–0.77 m/km, including long sequences exceeding 5 km (Mars3 series), indicating bounded long-term drift without explicit loop closure.

#### 6.4.1. Sequence-Level Cumulative Drift Analysis

**Figure 9:**
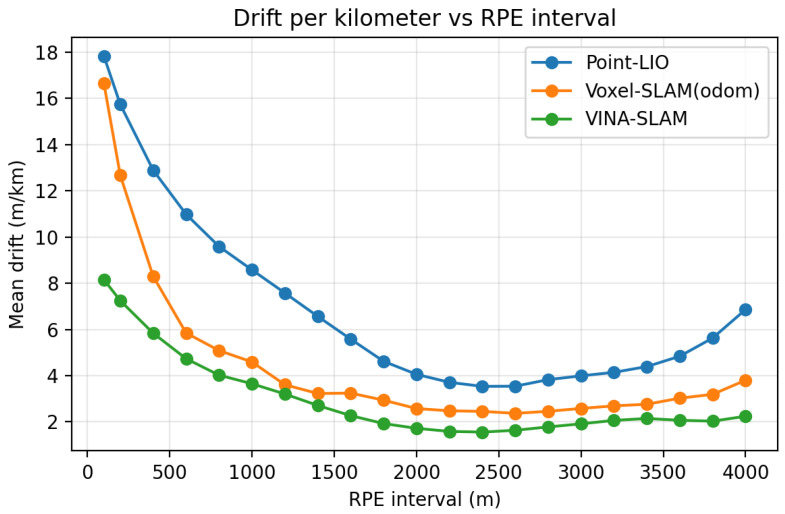
Mean drift (m/km) versus RPE interval length on the MARS-LVIG Mars4-1 sequence.

To further analyze long-term drift without loop closure at the sequence level, we report cumulative APE versus traveled distance and interval-dependent drift rate on Mars4-1 (Figure 9) under the same evaluation protocol described in Section 6.1. These sequence-level curves characterize within-trajectory error accumulation behavior. The degradation region is around 3000 m; after that the accumulated error begins to increase gradually, while before that it tends to be flat, indicating that our method can to some extent mitigate the accumulation of accumulated error. They complement (rather than replace) the dataset-level distance-normalized ATE (RMSE) reported in Table 4.

#### 6.4.2. Failure Cases and Current Limitations

Although VINA-SLAM improves accuracy on most sequences, it is not uniformly best across all conditions. In Table 1, Ours (odom + BA) is not the best on hilti06 (1.04 cm vs. 0.9 cm for Point-LIO), hilti07 (19.9 cm vs. 11.42 cm for Voxel-SLAM (odom)), and hilti11 (3.11 cm vs. 2.7 cm for Faster-LIO). A similar trend appears in Table 2 (hilti06, hilti07, and hilti11). These cases suggest several remaining limitations of voxel-map-based modeling: (i) a fixed voxel resolution introduces a trade-off between geometric fidelity and robustness under sparse long-range observations; (ii) aggressive rotations and rapidly changing viewpoints can temporarily reduce reliable voxel-plane correspondences; and (iii) when local coplanarity assumptions are violated, voxel-plane aggregation can accumulate bias. These limitations motivate future work on adaptive-resolution mapping, uncertainty-aware plane aggregation, and stronger outlier rejection under abrupt motion.

### 6.5. Performance Testing

Among the eight sequences of the HILTI 2022 dataset, three representative scene types are covered: corridors, outdoor areas, and indoor environments. Therefore, for the 25-sequence dataset used in this paper, we report the average computation time and memory consumption based solely on the HILTI 2022 sequences. All LiDAR scans are processed at a frequency of 10 Hz. In addition, memory usage serves as an important metric, as the system must store all necessary information in memory to support long-term data association and retrieval. As shown in Table 5, even on resource-limited industrial PCs (typically equipped with 8 GB of RAM), the memory consumption remains below the physical limit in most scenarios.

## 7. Conclusions and Future Works

This paper proposed VINA-SLAM, a tightly coupled LiDAR–Inertial SLAM framework to mitigate pose drift in geometrically degenerate or repetitive environments (e.g., long corridors and stairwells), where rotational observability and data association are often unreliable. The core contribution is the Voxel Normal Consistency (VNC) constraint, which injects rotation-sensitive geometric cues into both the front-end odometry and the sliding-window back-end optimization, thereby improving stability along degenerate directions and reducing drift. Extensive evaluations on 25 sequences from the HILTI and MARS-LVIG datasets show that VINA-SLAM achieves lower ATE on the majority of sequences compared with representative open-source baselines (e.g., FAST-LIO2 and Point-LIO), while remaining suitable for real-time deployment. On the 10 Hz HILTI sequences 01–08, the end-to-end latency stays within the LiDAR scan interval and the memory footprint remains bounded (Table 5).

Future work will focus on (i) integrating loop closure and global pose-graph optimization using normal and structural cues to improve global consistency, (ii) extending the current planar modeling to richer primitives (e.g., lines and cylinders) or learning-assisted residuals for strongly unstructured regions, and (iii) incorporating visual and semantic information to further enhance robustness under extreme degeneracy.

## Figures and Tables

**Table 1:** Comparison of translation RMSE on Hilti sequences (cm). ‘-’ indicates that the system diverged. The best results are highlighted in **bold**.

Sequences (cm)	hilti01	hilti02	hilti03	hilti04	hilti05	hilti06	hilti07	hilti08	hilti09	hilti10	hilti11	hilti12	hilti13
LeGO-LOAM	9.1	4.7	-	25.3	-	67	-	25.3	12.7	14.3	27.1	25.3	-
LiLi-OM	6.2	22.2	-	31.0	-	28.9	-	20.3	6.9	8.5	19.9	31	-
LINS	6.5	18.8	-	20.7	-	23.1	-	17.8	7.5	9.9	28.1	20.7	-
LIO-SAM	7.4	15.2	-	23.4	-	17.4	-	22.4	6.6	6.8	17.6	23.4	74
FAST-LIO2	1.3	2.8	32.0	6.7	55	2.4	72	**1.7**	2.4	1.8	4.2	6.7	16
Faster-LIO	1.1	2.1	37.0	5.0	73	1.4	61	2.4	1.9	2.3	**2.7**	5.0	11.4
Point-LIO	1.1	3.0	23.0	3.7	44	**0.9**	45	2.6	3.2	1.6	3.6	3.7	9.2
Voxel-SLAM (odom)	0.89	2.5	4.42	5.41	15.68	1.73	**11.42**	5.16	1.6	2.0	2.8	5.41	4.3
Ours (odom)	0.86	2.01	3.75	5.39	15.62	1.5	21.31	5.11	1.53	1.81	3.52	3.37	3.26
**Ours (odom + BA)**	**0.71**	**1.8**	**3.09**	**3.36**	**15.49**	1.04	19.9	1.96	**1.45**	**1.37**	3.11	**1.11**	**1.27**

**Table 2:** Distance-normalized ATE (RMSE) comparison on HILTI dataset (m/km). ‘-’ indicates that the system diverged. The best results are highlighted in **bold**.

Method	hilti01	hilti02	hilti03	hilti04	hilti05	hilti06	hilti07	hilti08	hilti09	hilti10	hilti11	hilti12	hilti13
LeGO-LOAM	0.569	0.152	-	2.30	-	8.38	-	1.81	0.747	0.953	1.81	0.904	-
LiLi-OM	0.388	0.716	-	2.82	-	3.61	-	1.45	0.406	0.567	1.33	1.11	-
LINS	0.406	0.606	-	1.88	-	2.89	-	1.27	0.441	0.660	1.87	0.739	-
LIO-SAM	0.463	0.490	-	2.13	-	2.18	-	1.60	0.388	0.453	1.17	0.836	5.29
FAST-LIO2	0.0813	0.0903	1.39	0.609	2.89	0.300	5.54	0.121	0.141	0.120	0.280	0.239	1.14
Faster-LIO	0.0688	0.0677	1.61	0.455	3.84	0.175	4.69	0.171	0.112	0.153	0.180	0.179	0.814
Point-LIO	0.0688	0.0968	1.00	0.336	2.32	0.113	3.46	0.186	0.188	0.107	0.240	0.132	0.657
Voxel-SLAM (odom)	0.0556	0.0806	0.192	0.492	0.825	0.216	0.878	0.369	0.0941	0.133	0.187	0.193	0.307
Ours (odom)	0.0538	0.0648	0.163	0.490	0.822	0.188	1.64	0.365	0.0900	0.121	0.235	0.120	0.233
**Ours (odom + BA)**	**0.0444**	**0.0581**	**0.134**	**0.305**	**0.815**	0.130	1.53	**0.140**	**0.0853**	**0.0913**	0.207	**0.0396**	**0.0907**

**Table 3:** Absolute trajectory error (RMSE, m) of different odometry and SLAM methods on the MARS-LVIG dataset. ‘-’ indicates that the system diverged. The best results are highlighted in **bold**.

Sequences (m)	Mars1-1	Mars1-2	Mars1-3	Mars2-1	Mars2-2	Mars2-3	Mars3-1	Mars3-2	Mars3-3	Mars4-1	Mars4-2	Mars4-3
LiLi-OM	4.56	4.81	5.35	3.68	3.72	3.70	10.54	11.66	13.08	-	-	-
LIO-SAM	3.77	4.02	4.79	1.22	1.30	1.39	6.89	6.95	8.62	12.09	11.53	14.64
FAST-LIO2	0.66	0.46	0.48	0.26	0.39	0.59	2.17	2.05	2.51	4.46	6.54	8.37
Faster-LIO	0.42	0.56	0.51	0.27	0.36	0.32	1.71	1.49	3.12	5.50	7.43	8.79
Point-LIO	1.53	1.44	1.47	0.35	0.40	0.69	3.63	3.38	4.23	8.92	9.57	12.45
Voxel(odom)	0.45	0.42	0.50	0.24	0.42	0.48	2.25	1.39	1.90	4.21	5.33	7.68
Ours (odom)	0.41	0.43	0.51	0.19	0.24	0.36	1.17	1.35	1.57	2.84	3.55	3.74
**Ours (odom + BA)**	**0.25**	**0.29**	**0.33**	**0.13**	**0.17**	**0.22**	**0.99**	**1.12**	**1.33**	**2.39**	**2.75**	**3.30**

**Table 4:** Distance-normalized ATE (RMSE) comparison on MARS-LVIG dataset (m/km). ‘-’ indicates that the system diverged. The best results are highlighted in **bold**.

Method	Mars1-1	Mars1-2	Mars1-3	Mars2-1	Mars2-2	Mars2-3	Mars3-1	Mars3-2	Mars3-3	Mars4-1	Mars4-2	Mars4-3
LiLi-OM	2.46	2.60	2.89	1.80	1.82	1.81	2.06	2.28	2.56	-	-	-
LIO-SAM	2.04	2.17	2.59	0.598	0.637	0.681	1.35	1.36	1.69	2.81	2.68	3.40
FAST-LIO2	0.357	0.249	0.259	0.127	0.191	0.289	0.425	0.401	0.491	1.04	1.52	1.95
Faster-LIO	0.227	0.303	0.276	0.132	0.176	0.157	0.335	0.292	0.611	1.28	1.73	2.04
Point-LIO	0.827	0.778	0.795	0.172	0.196	0.338	0.711	0.661	0.828	2.07	2.22	2.90
Voxel-SLAM (odom)	0.243	0.227	0.270	0.118	0.206	0.235	0.440	0.272	0.372	0.979	1.24	1.79
Ours (odom)	0.222	0.232	0.276	0.0931	0.118	0.176	0.229	0.264	0.307	0.661	0.826	0.870
**Ours (odom + BA)**	**0.135**	**0.157**	**0.178**	**0.0637**	**0.0833**	**0.108**	**0.194**	**0.219**	**0.260**	**0.556**	**0.640**	**0.767**

**Table 5:** Time and memory consumption of HILTI 2022 sequences 01–08.

(Per Scan)	hilti01	hilti02	hilti03	hilti04	hilti05	hilti06	hilti07	hilti08
Odometry (ms)	66.527	50.267	15.618	10.676	12.267	22.465	16.631	33.347
Local BA (ms)	20.130	2.868	1.724	1.172	1.826	5.288	3.789	7.152
Total time (ms)	86.657	57.165	17.342	11.848	14.093	27.753	20.420	40.499
Total memory usage (GB)	4.980	4.404	1.186	0.550	1.131	1.101	1.069	1.657

## Data Availability

The data presented in this study are openly available in VINA-SLAM https://github.com/SheepYang666/VINA-SLAM accessed on 9 March 2026.

## Appendix A. Iterative Optimization for LiDAR-Inertial BA

During the local BA optimization, the cost function within the sliding window is formulated as Equation (13). Given the perturbation of the state X, denoted as $\delta X=[\delta x_{1},\delta x_{2},\ldots ,\delta x_{N},\delta g]$:

(A1) $$\begin{array}{c} c\left(X⊞\delta X\right)=\arg\min_{X}\left(\frac{1}{2}\sum_{i=1}^{N-1}{∥r_{i,i+1}\left(X\right)∥}_{\Sigma_{i,i+1}^{-1}}^{2}\right.\\ \left.\;\;+\sum_{j=1}^{M}c_{j}\lambda_{j}^{\min}\left(X\right)+\frac{1}{2}\sum_{j=1}^{M}\alpha_{j}{∥r_{j}\left(X\right)∥}^{2}\right) \end{array}$$

The residuals are expanded to the second-order approximation as:

(A2) $$\begin{array}{cc} c(X⊞\delta X)\approx & \frac{1}{2}\sum_{i=1}^{N-1}\left(∥r_{i,i+1}{\left(X\right)∥}_{\Sigma_{i,i+1}^{-1}}^{2}+2r_{i,i+1}{\left(X\right)}^{⊤}\Sigma_{i,i+1}^{-1}J_{i}\;\delta X+\delta X^{⊤}J_{i}^{⊤}\Sigma_{i,i+1}^{-1}J_{i}\;\delta X\right)\\ \; & +\sum_{j=1}^{M}\left(c_{j}\lambda_{j}^{\min}\left(X\right)+c_{j}\;g_{j}^{⊤}\delta X+\frac{1}{2}c_{j}\;\delta X^{⊤}H_{j}\;\delta X\right)\\ \; & +\frac{1}{2}\sum_{j=1}^{M}\left(\alpha_{j}{∥r_{j}∥}^{2}+2\alpha_{j}r_{j}^{⊤}J_{j}\;\delta X+\alpha_{j}\;\delta X^{⊤}J_{j}^{⊤}J_{j}\;\delta X\right)\\ = & \sum_{j=1}^{M}c_{j}\lambda_{j}^{\min}\left(X\right)+\frac{1}{2}\sum_{j=1}^{M}\alpha_{j}∥r_{j}{∥}^{2}+\frac{1}{2}\sum_{i=1}^{N-1}{∥r_{i,i+1}\left(X\right)∥}_{\Sigma_{i,i+1}^{-1}}^{2}\\ \; & +\frac{1}{2}\delta X^{⊤}\left(\sum_{j=1}^{M}\left(c_{j}\;H_{j}+\alpha_{j}J_{j}^{⊤}J_{j}\right)+\sum_{i=1}^{N-1}J_{i}^{⊤}\Sigma_{i,i+1}^{-1}J_{i}\right)\delta X\\ \; & +\left(\sum_{j=1}^{M}{\left(c_{j}\;g_{j}+\alpha_{j}J_{j}^{⊤}r_{j}\right)}^{⊤}+\sum_{i=1}^{N-1}r_{i,i+1}{\left(X\right)}^{⊤}\Sigma_{i,i+1}^{-1}J_{i}\right)\delta X \end{array}$$

Here, $g_{j}$ and $H_{j}$ denote the gradient and Hessian matrix of the minimum eigenvalue term, respectively. $r_{j}=\left(I-n_{j}^{\mathrm{map}}n_{j}^{{\mathrm{map}}^{⊤}}\right)u_{0}^{\left(j\right)}$ represents the normal-alignment residual, $J_{j}$ is its corresponding Jacobian matrix, the approximate Hessian is given by $J_{j}^{⊤}J_{j}$, and $\alpha_{j}$ denotes the weighting coefficient of the rotational factor constraint.

## Appendix B. Details of the Dataset

The detailed information of all 25 sequences mentioned in Section 6 is provided in Table A1.

## Appendix C. Degradation Detection

To determine whether the current geometry exhibits observability degradation, we perform spectral analysis on the matrix constructed from normal-vector observations and use the minimum eigenvalue as the evaluation criterion.

Let the set of unit normals after sign disambiguation for the current frame be denoted as ${\left\{n_{i}\right\}}_{i=1}^{M}$ ($∥n_{i}∥=1$). Then,

(A3) $$N\;≜\;\sum_{i=1}^{M}n_{i}\;n_{i}^{⊤}\;\in \;R^{3\times 3}.$$

Intuitively, N aggregates the constraint strength of the current frame across different rotational directions: if the normals are uniformly distributed, all eigenvalues of N are relatively large; whereas if the normals are concentrated along a few directions, certain degrees of freedom will exhibit significant information deficiency. By performing PCA on N, the minimum eigenvalue $\lambda_{\min}$ represents the weakest constraint direction (e.g., rotation about the normal axis in a long corridor). Let $T_{d}$ denote the per-frame degeneration threshold. A frame is classified as degenerate if $\lambda_{\min}<T_{d}$; otherwise, it is regarded as sufficiently observable.

**Table A1:** Details of all the sequences for experiments.

Sequence	Name	Duration (min:s)	Distance (km)
hilti01	exp01-construction	3:47	0.16
hilti02	exp02-construction	7:10	0.31
hilti03	exp03-construction	5:09	0.23
hilti04	exp07-long-corridor	2:12	0.11
hilti05	exp09-cupola	7:26	0.19
hilti06	exp11-lower-gallery	2:31	0.08
hilti07	exp15-upper-gallery	4:20	0.13
hilti08	exp21-outside	2:32	0.14
hilti09	site1-handheld-1	3:24	0.17
hilti10	site1-handheld-2	2:47	0.15
hilti11	site1-handheld-3	2:50	0.15
hilti12	site1-handheld-4	4:55	0.28
hilti13	site1-handheld-5	2:39	0.14
mars1-1	HKisland01	9:40	1.85
mars1-2	HKisland02	5:10	1.85
mars1-3	HKisland03	3:46	1.85
mars2-1	HKairport01	10:50	2.04
mars2-2	HKairport02	5:40	2.04
mars2-3	HKairport03	4:00	2.04
mars3-1	AMtown01	20:00	5.11
mars3-2	AMtown02	10:10	5.11
mars3-3	AMtown03	7:10	5.11
mars4-1	AMvalley01	17:00	4.30
mars4-2	AMvalley02	8:45	4.30
mars4-3	AMvalley03	6:00	4.30
**Total**		159:53	42.14
